# Invasive Pneumococcal Disease in a Patient With COVID-19: A Case Report

**DOI:** 10.7759/cureus.13559

**Published:** 2021-02-25

**Authors:** Sarah Ayad, Ramez Alyacoub, Kirolos Gergis, Daniel Grossman, Julius Salamera

**Affiliations:** 1 Internal Medicine, Rutgers-New Jersey Medical School/Trinitas Regional Medical Center, Elizabeth, USA; 2 Internal Medicine, McLaren Flint Hospital, Flint, USA; 3 Infectious Disease, Rutgers-New Jersey Medical School/Trinitas Regional Medical Center, Elizabeth, USA

**Keywords:** covid-19, sars cov 2, streptococcus pneumoniae, invasive pneumococcal disease

## Abstract

The spread of the new severe acute respiratory syndrome coronavirus 2 (SARS-CoV-2), which causes coronavirus disease 2019 (COVID-19), has resulted in a global health pandemic and caused profound morbidity and mortality worldwide. The virus is known to cause severe hypoxemic respiratory failure and has been associated with extrapulmonary manifestations and end-organ dysfunction in the setting of extensive inflammatory response. Recently, the association between COVID-19 and pneumococcal pneumonia co-infection or superinfections has gained increasing interest. In this report, we present the case of a 58-year-old man with a past medical history significant for pulmonary tuberculosis, diagnosed over two decades ago, who presented with pleuritic chest pain, myalgia, intermittent fevers, chills, and productive cough and was found to have invasive pneumococcal disease and COVID-19. To our knowledge, this is the first reported case of invasive pneumococcal infection in a patient with COVID-19.

## Introduction

Severe acute respiratory syndrome coronavirus 2 (SARS-CoV-2), which causes coronavirus disease 2019 (COVID-19), was first reported in China in December 2019 following what was described as a widespread incidence of viral pneumonia in the city of Wuhan [1]. COVID-19 has caused over 100 million infections and more than two million deaths globally as of January 2021 [2]. The virus accesses the body using angiotensin-converting enzyme 2 (ACE2) receptors, which are found in high levels in lung tissue (as well as the heart, ileum, kidney, and bladder), often causing severe respiratory disease [3]. COVID-19 is known to cause multi-organ failure as well as thrombosis and pulmonary embolism in its severe manifestations [3]. The specific mechanisms behind COVID-19 causing significant morbidities such as multi-organ failure and thrombosis are still being studied by researchers.

Historically, the occurrence of concomitant or secondary bacterial infection in persons with viral respiratory tract infection has been associated with poor prognosis and poor outcomes. For instance, in patients with influenza, 20-30% suffer from superimposed bacterial infections [4]. The existence of superimposed bacterial infections in these patients has been associated with increased risks of mechanical ventilation and death. While the prevalence of superimposed or bacterial infections in COVID-19 patients is not yet well known, in one meta-analysis, bacterial co-infections were reported in 3.5% of COVID-19 patients, and superimposed bacterial infections were seen in 15.5% [4]. Complicating bacteremia in itself accounts for an even smaller percentage of bacterial infections associated with COVID-19. The presence of bacterial infection, with or without hematogenous involvement, affects the management of the condition, and antibiotic regimens have to be tailored to prevent the emergence of resistance over time. In light of this, we report the case of a 58-year-old male, with a past medical history significant for pulmonary tuberculosis, who was found to have bacteremic Streptococcus pneumoniae pneumonia and severe COVID-19 infection.

## Case presentation

Our patient was a 58-year-old male with a past medical history of pulmonary tuberculosis diagnosed over two decades ago; he was brought to the emergency department in March 2020, complaining of less than a week's history of chest pain. The pain was sharp, pleuritic in nature, left-sided, and associated with myalgia, intermittent fevers, chills, and productive cough. The patient also endorsed exposure to SARS-CoV-2. He denied any palpitations, dizziness, syncopal episodes, lightheadedness, abdominal pain, vomiting, or nausea. The patient denied any history of immunosuppressive condition, functional/anatomic asplenia, or chronic kidney/cardiovascular/lung disease. On presentation, the patient was normotensive (blood pressure: 120/70 mmHg), tachycardic (heart rate: 106 beats per minute), with oxygen saturation of 95% on room air, and a temperature of 99.9 °F. Physical examination revealed an age-appropriate male on oxygen supplementation via nasal cannula who was in no apparent respiratory distress. Lung examination showed the presence of crackles on the left upper lung fields and extending to the left mid-lung field as well. The rest of the physical exam was unremarkable.

**Figure 1:**
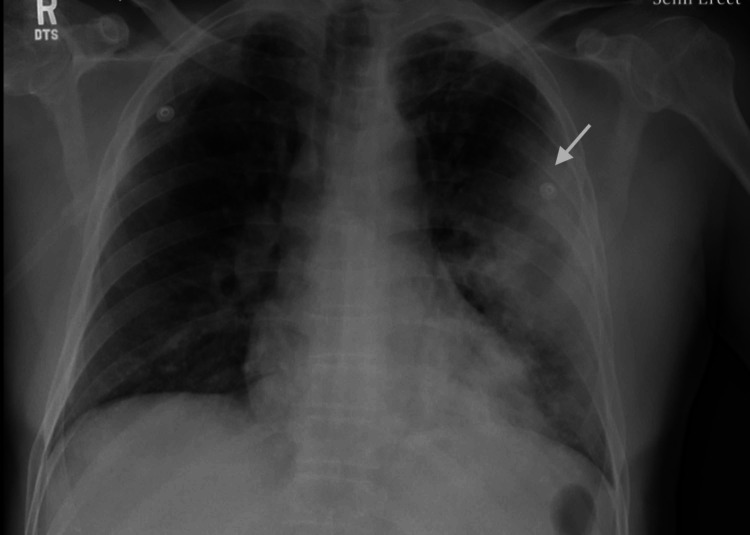
Initial CXR of the patient The image shows large airspace consolidation in the left mid-lung with a cavitary appearance (arrow), and bi-apical reticular opacities, more prominent on the left than right CXR: chest X-ray

**Figure 2:**
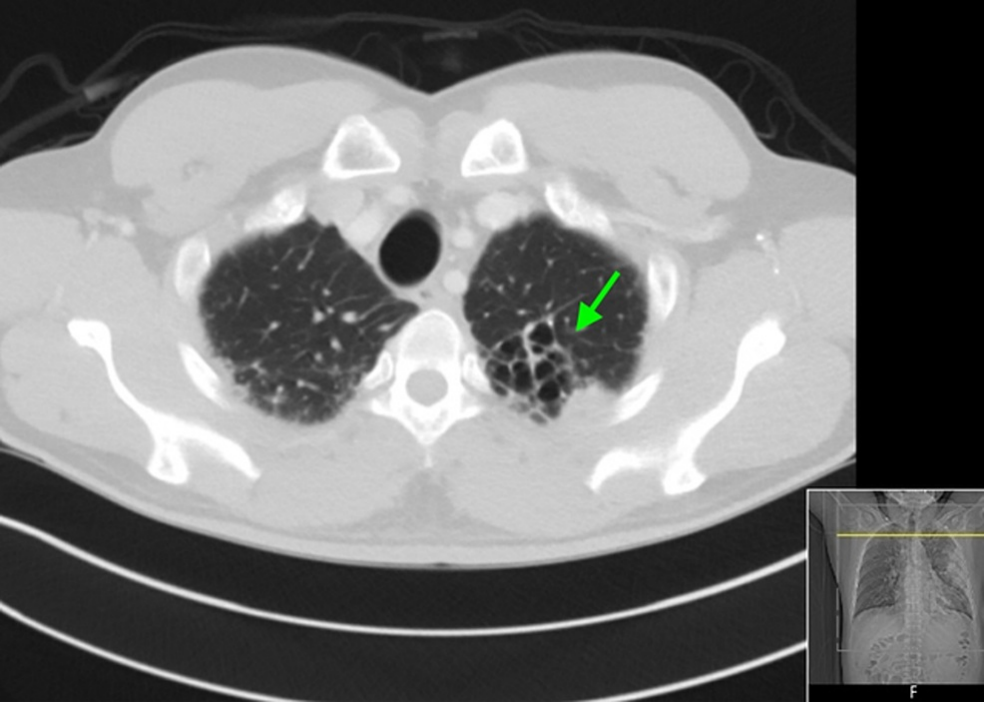
CT of the chest - image 1 The image shows evidence of mediastinal adenopathy and cystic bronchiectasis in the left upper lobe with calcification suggesting old granulomatous disease (arrow) CT: computed tomography

**Figure 3:**
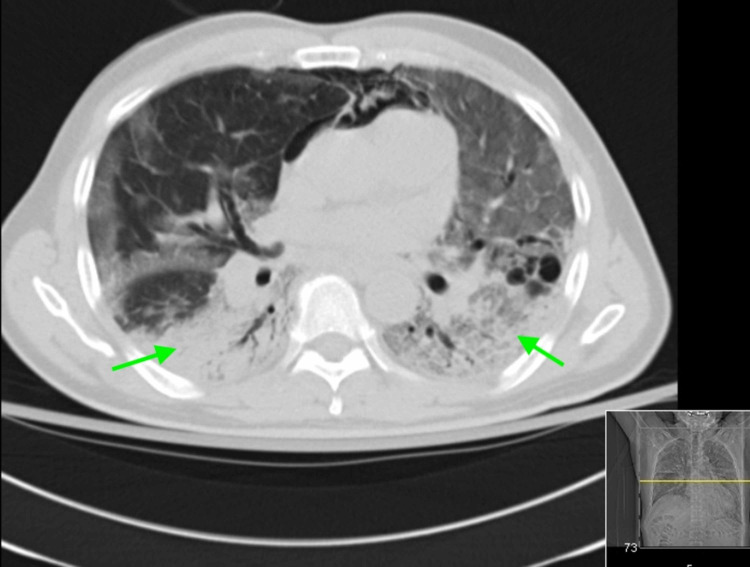
CT of the chest - image 2 The image shows ground-glass airspace opacities bilaterally (arrows) CT: computed tomography

**Figure 4:**
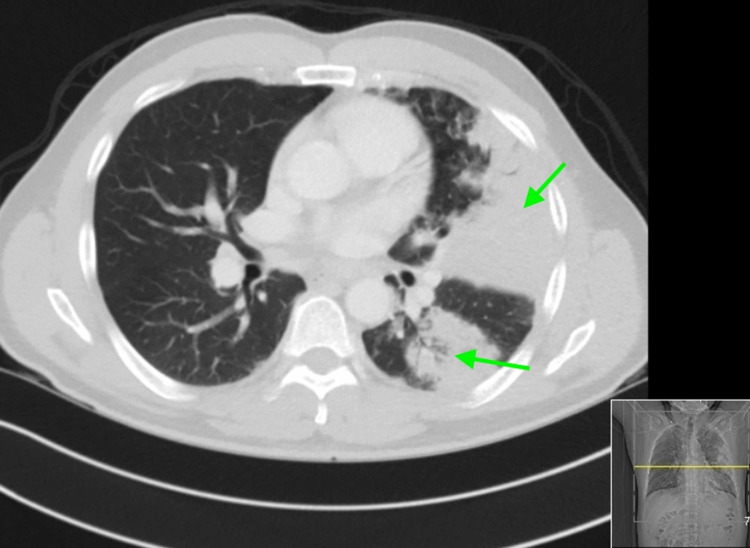
CT of the chest - image 3 The image shows dense areas of pulmonary consolidation particularly involving the left upper lobe with the consolidation of at least half of the left upper lobe (arrows) CT: computed tomography

Diagnostic test results on presentation are outlined in Table 1. A nasopharyngeal swab for influenza A and B polymerase chain reaction (PCR) were negative, and a pneumococcal urinary antigen was positive. Chest X-ray (CXR) showed large airspace consolidation in the left mid-lung with a cavitary appearance and bi-apical reticular opacities, more prominent on the left than the right, as seen in Figure 1. Ultimately, a nasopharyngeal swab for real-time reverse-transcription polymerase chain reaction (rRT-PCR) returned positive for SARS-CoV-2. CT of the chest showed evidence of mediastinal adenopathy and cystic bronchiectasis in the left upper lobe with calcification suggestive of old granulomatous disease, as seen in Figure 2. There were also multiple ground-glass airspace opacities bilaterally in combination with dense areas of pulmonary consolidation particularly involving the left upper lobe, with the consolidation of at least half of the left upper lobe (Figure 3, Figure 4). An electrocardiogram on presentation demonstrated a coved ST-segment elevation in V1-V2, suggesting Brugada pattern type 1 without evidence of ischemic changes.

**Table 1:** Laboratory values upon presentation to the ED ED: emergency department

Variables	Reference range	Values on presentation
White blood count	4.8–10.8 K/uL	10.3
Hemoglobin	14–18 gm/dL	12.9
Platelet	130 K–400 K	140
Polys	42–75%	93.2
Polys (absolute)	1.4–6.5 K/uL	9.6
Lymphocytes	20.5–51.1%	4.9
Absolute lymphocytes	1.2–3.4 K/uL	0.5
Lactic acid	0.5–2.2 mmol/L	1.4
Sodium	136–144 mmol/L	116
Potassium	3.6–5.1 mmol/L	3.8
Creatinine	0.7–1.2 mg/dL	0.81

The patient was admitted to the general medical floors and started on empiric ceftriaxone, azithromycin, and hydroxychloroquine. The following day, two sets of blood cultures were suggestive of gram-positive cocci in chains and pairs on preliminary Gram stain, which was later identified as Streptococcus pneumoniae, penicillin-susceptible, by VITEK automated blood culture system (BioMérieux SA, Marcy-l'Étoile, France). At that time, azithromycin was discontinued and the ceftriaxone dose was adjusted to 2 g IV daily. The patient received hydroxychloroquine for 10 days and IV ceftriaxone for 18 days. Repeat blood cultures revealed no growth. Rapid HIV was non-reactive. Oxygen supplementation through nasal cannula was started, but eventually, the patient required non-invasive positive pressure ventilation (CPAP) due to worsening hypoxemia. Two weeks into hospitalization, his condition deteriorated, and he ended up on mechanical ventilatory support due to hypoxemic respiratory failure.

**Figure 5:**
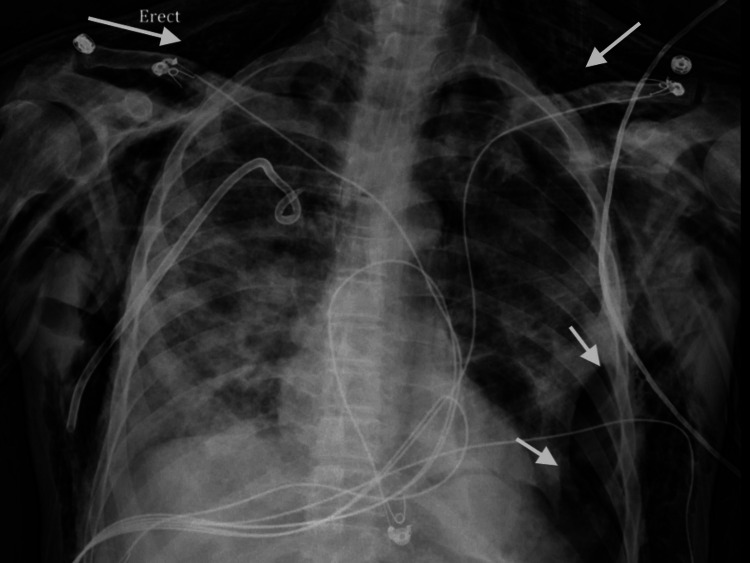
Repeat CXR during the hospital course The image shows small-to-moderate-sized left basilar pneumothorax and diffuse subcutaneous emphysema (arrows) CXR: chest X-ray

On week six of hospitalization, the patient received one unit of convalescent (COVID-19-rich) plasma. The hospital course was complicated by hemodynamically unstable atrial flutter requiring synchronized cardioversion, severe barotrauma necessitating chest tube placement, and severe subcutaneous emphysema, as seen in Figure 5. Additionally, several complicating infections including bacteremic methicillin-susceptible Staphylococcus aureus (MSSA) pneumonia, Candida albicans fungemia, and Enterobacter cloacae lower respiratory tract infection were seen. The patient’s condition continued to worsen and on day 54 of hospitalization, he went into asystolic cardiac arrest. Full advanced cardiac life support (ACLS) protocol was performed; however, unfortunately, the patient died.

## Discussion

Streptococcus pneumoniae is a gram-positive coccus and is the most common cause of community-acquired bacterial pneumonia. It can cause non-invasive diseases such as community-acquired pneumonia and otitis media. Invasive pneumococcal disease is defined as the identification of bacteria in sterile body fluids such as cerebrospinal fluid (CSF) (i.e., in meningitis) or blood, (i.e., bacteremia) [5]. Pneumococcal bacteremia can occur as a result of pneumococcal pneumonia or in its absence. When bacteremia is present, secondary complications such as arthritis, meningitis, and endocarditis may occur. Risk factors for invasive pneumococcal disease include advanced or very young age (elderly population and young children), immunosuppression from HIV, liver disease, renal disease, diabetes mellitus, smoking, functional as well as anatomic asplenia, and history of hematologic malignancies such as multiple myeloma [6].

The risk of invasive pneumococcal disease seems to be associated with viral respiratory illnesses, such as influenza. A temporal association between invasive pneumococcal disease and exposure to common respiratory viruses during winter months was observed in a prospective study of 4,147 invasive pneumococcal disease episodes by Talbot et al. The study concluded that the average weekly frequency of invasive pneumococcal disease during respiratory syncytial virus (RSV) and influenza seasons was higher than during the nonviral seasons [7].

Several cases of COVID-19 and pneumococcal pneumonia co-infection or superinfections have been reported since the emergence of COVID-19 as a global pandemic [8]. However, this is the first reported case of invasive pneumococcal disease in association with COVID-19 infection. Diagnosing secondary bacterial infections and co-infections is challenging as many of the features tend to overlap. Findings that increase the likelihood of bacterial infection, which may warrant empiric antibiotic therapy pending culture results, include new leukocytosis, particularly neutrophilic leukocytosis, and new-onset fever [9]. CXR findings of lobar consolidation may also be a clue. Urine pneumococcal antigen is a sensitive test to diagnose pneumococcal pneumonia. Positive blood culture indicates invasive disease, as was the case in our patient.

Bacteremia is considered a risk factor for more unsatisfactory outcomes or complications in patients with pneumococcal pneumonia in the absence of critical illness [10-11]. Bacteremic pneumococcal community-acquired pneumonia is associated with higher in-hospital mortality; patients typically have a longer length of stay compared to those with non-bacteremic pneumococcal pneumonia [12]. In a study by Bordon et al. involving 833 pneumococcal community-acquired pneumonia patients, higher plasma levels of C-reactive protein (CRP), procalcitonin, and brain natriuretic peptide (BNP) were found in bacteremic than in non-bacteremic patients. The bacteremic group had consistently higher plasma levels of both pro-and anti-inflammatory cytokines [10].

Recommendations regarding the choice of therapy for bacteremic versus non-bacteremic pneumococcal pneumonia do not generally differ from each other. Observational studies suggest that outcomes for sicker patients with bacteremic pneumococcal pneumonia might be improved with combination therapy that includes a beta-lactam and a macrolide, or a respiratory fluoroquinolone [13]. The addition of macrolide antibiotics may play a beneficial role due to its well-documented anti-inflammatory effects [14].

Our patient was treated for pneumococcal pneumonia; however, his respiratory condition deteriorated because of COVID-19 infection, and he eventually required mechanical ventilation. He had a long and complicated course in the intensive care unit and, unfortunately, did not survive. To our knowledge, this is the first reported case of bacteremic pneumococcal pneumonia with COVID-19 infection. More studies are needed to study the association between bacteremic pneumococcal pneumonia and novel coronavirus infection.

## Conclusions

In over a year, COVID-19 has caused massive resource constraints around the world and led to many detrimental consequences. In the current literature, a number of cases of COVID-19 and pneumococcal pneumonia co-infection have been reported. However, cases of bacteremic pneumococcal pneumonia with COVID-19 are uncommon in the hospital setting, and our case report adds to the limited literature available. Bacterial infection in a COVID-19 patient warrants targeted antibiotic therapy in addition to other available COVID-19-specific therapeutics. If pneumococcal bacteremic pneumonia is suspected in a COVID-19 patient, the regimen of a beta-lactam and a macrolide, or a respiratory fluoroquinolone can be considered. Further studies are essential to examine and clarify the association between bacteremic pneumococcal pneumonia and COVID-19.
